# Self-Healable and Super-Tough Double-Network Hydrogel Fibers from Dynamic Acylhydrazone Bonding and Supramolecular Interactions

**DOI:** 10.3390/gels8020101

**Published:** 2022-02-08

**Authors:** Jiachuan Hua, Chang Liu, Bin Fei, Zunfeng Liu

**Affiliations:** 1 Institute of Textiles and Clothing, The Hong Kong Polytechnic University, Hong Kong 999077, China; jc.hua@connect.polyu.hk (J.H.); judyc.liu@connect.polyu.hk (C.L.); 2 Key Laboratory of Functional Polymer Materials, College of Chemistry, Nankai University, Tianjin 300071, China; liuzunfeng@nankai.edu.cn

**Keywords:** hydrogel fiber, iota carrageenan, dynamic covalent bond, double network, self-healing

## Abstract

Macroscopic hydrogel fibers are highly desirable for smart textiles, but the fabrication of self-healable and super-tough covalent/physical double-network hydrogels is rarely reported. Herein, copolymers containing ketone groups were synthesized and prepared into a dynamic covalent hydrogel via acylhydrazone chemistry. Double-network hydrogels were constructed via the dynamic covalent crosslinking of copolymers and the supramolecular interactions of iota-carrageenan. Tensile tests on double-network and parental hydrogels revealed the successful construction of strong and tough hydrogels. The double-network hydrogel precursor was wet spun to obtain macroscopic fibers with controlled drawing ratios. The resultant fibers reached a high strength of 1.35 MPa or a large toughness of 1.22 MJ/m^3^. Highly efficient self-healing performances were observed in hydrogel fibers and their bulk specimens. Through the simultaneous healing of covalent and supramolecular networks under acidic and heated conditions, fibers achieved rapid and near-complete healing with 96% efficiency. Such self-healable and super-tough hydrogel fibers were applied as shape memory fibers for repetitive actuating in response to water, indicating their potential in intelligent fabrics.

## 1. Introduction

Hydrogels are soft materials with a crosslinked network structure. Macroscopic fibers developed from functional hydrogels are ideal candidates for smart textiles (e.g., fibrous actuators [1], hygiene textiles [2] and flexible sensors [3,4,5]). However, hydrogel fibers have undesirable mechanical weaknesses and brittleness, which have limited their real uses, as has the complexity of processing flowable dope into crosslinked gel strands. In our previous work, hydrogels with high toughness have been synthesized and applied in cell cultures [6], shape memory actuators [7,8,9] and intelligent textiles [2,10]. Based on dynamic covalent bonding and the self-assembly of polysaccharide networks, self-healable hydrogel fibers with mechanical robustness would be attractive for the further development of hydrogel textiles.

Improvements on their mechanical properties have been focused on the hydrogel area. Recent strategies include mechanochemical self-strengthening (e.g., self-growing [11], strain-induced crystallization [12] and stored length releasing [13]), the introduction of sacrificial structures (e.g., interpenetrating network [9,14] and supramolecular interaction [15,16]) and the prevention of crack propagation (e.g., nanocomposites [17,18,19] and macromolecular microsphere composites [20,21]). Among these strategies, double-network (DN) structures have been the most successful strategy to construct strong and tough hydrogels since the pioneering work was conducted in 2003 [22]. By synthesizing stiff and ductile networks simultaneously or sequentially, energy could be dissipated in such hydrogels during mechanical deformation to avoid destructive stress concentration on polymer strands. Based on polymerized covalent networks and physically crosslinked polysaccharide networks, hybrid DN hydrogels have exhibited ultra-high stretchability and toughness that makes them suitable for fiber spinning [8,23,24]. However, the irreversible destruction of covalent networks in such hydrogels is gradually intensified during cyclic deformation and has led to fatigue fracture [25]. To avoid the embrittling caused by fractured polymer strands, the development of a self-healable network is essential for durable and reliable hydrogel fibers.

Self-healable networks rely on the reversibility of crosslinks. Physical networks in DN hydrogels are usually crosslinked via supramolecular interactions, which were widely found to be self-repairable. Typical examples include hydrogen bonding-induced helix bundle assembly (e.g., carrageenan [9], agar [26], gelatin [27] and DNA [28]), electrostatic attraction (e.g., negatively charged groups and multivalent cations [23], poly ionic liquids and negatively charged crosslinkers [29]) and aggregation domains (e.g., semi-crystallinity [30] and hydrophobic interaction [31]). In DN hydrogels, the major challenge is the recovery of damaged covalent networks. Dynamic covalent crosslinks are desirable to achieve complete healing in DN structures, which involves the exchanging of the reaction partner or the formation of new dynamic covalent bonds. The regeneration of dynamic covalent bonds usually requires a trigger from external stimuli. Recently reported examples include the disulfide exchange reaction (redox-responsive) [32,33], imine condensation (pH-responsive) [34,35] and Diels–Alder chemistry (heat-responsive) [36]. By incorporating functional components to achieve the conversion of specific stimuli to other types (e.g., turning acoustic energy to heat), more self-healing hydrogels that are desirable for industry could be developed and applied in future.

The spinning of hydrogel has been challenging primarily due to the weakness of the nascent fiber and the difficulty of processing crosslinked networks where polymer chains are not flowable. The representative routes for constructing hydrogel fibers are ‘monomers to network’ and ‘macromolecules to network’. To overcome the low viscosity of monomer solutions, which leads to the breakage of nascent fiber, a precursor (containing monomers, initiators and crosslinkers) usually polymerizes in tubular templates, which are lubricated or super-hydrophobic [37]. Although fibrous hydrogels with millimeter levels of thickness (thin fibers are poorly demoldable) have been fabricated by such reactive spinning methods, its industrialization has encountered challenges to achieving high-speed manufacturing and obtaining fibers at the micrometer level [38]. In comparison, the more scalable spinning route in the textile industry is by constructing networks using macromolecules. As a facile method to obtain macroscopic hydrogel fibers, wet-spinning has utilized rapid physical interactions to solidify injected dope columns in coagulation baths (the use of covalent crosslinks has rarely been reported in recent literature [39]). Typical solidification strategies include constructing electrostatic attraction (e.g., -COO^−^ and multivalent cations [40] and -NH_3_^+^ and negatively charged groups [41]), hydrogen bonding (e.g., agarose double helixes [42] and collagen nanofibrils [43]) and aggregation (e.g., destroying colloidal stability [44] and dehydration by non-solvents [42]). The introduction of multiple and synergistic interactions to several biopolymers (e.g., carrageenan [45], agar [46] and gelatin [30]) offers toughening for nascent fibers. Via the simultaneous or sequential synthesis of covalent networks in physical crosslinked nascent fibers, DN hydrogel fibers are able to be fabricated and find wide applications [47].

In this study, acylhydrazone bonding was utilized for synthesizing a dynamic covalent hydrogel. By introducing a supramolecular network, self-healable DN hydrogels could be constructed with significantly enhanced tensile properties in contrast with their single network (SN) parents.

## 2. Results and Discussion

### 2.1. SN Hydrogels from Dynamic Covalent and Supramolecular Networks

**Figure 1:**
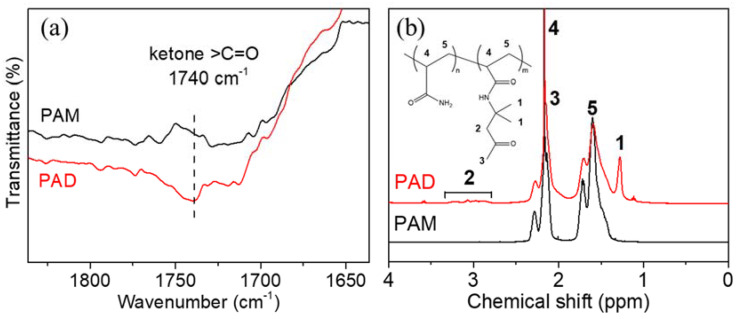
(**a**) FTIR and (**b**) ^1^H-NMR (inset image: structure of PAD) spectra of PAD-5 and PAM.

Prior to the preparation of DN hydrogels, the parental polyacrylamide-co-diacetone acrylamide (PAD) and iota-carrageenan (IC) single-network hydrogels were synthesized and studied. In order to construct dynamic covalent networks, PAD copolymers were synthesized via radical polymerization (reaction in Figure S1). At a temperature of 70 °C, acrylamide (AM) and diacetone acrylamide (DAAM) monomers were initiated by ammonium persulfate (APS), and randomly copolymerized into macromolecular chains. To provide the successful copolymerization, PAD-5 and parallelly synthesized polyacrylamide (PAM) were selected as typical examples for Fourier transform infrared spectroscopy (FTIR) (Figure 1a) and ^1^H-NMR (b) characterizations. The FTIR spectrum of PAD-5 shows a peak at 1740 cm^−1^ (absent in the PAM), which was assigned to ketone carbonyl groups (-CH_2_-CO-CH_3_). The ^1^H-NMR spectrum of PAD-5 showed new signals at 1.28 and 2.79–3.31 ppm, in contrast with that of PAM. This evidence proves the successful copolymerization of DAAM monomers. By calculating the integral area in the NMR curve (peak 1 = 33.5, peak 5 = 224.0), the molar percentage of DAAM was calculated as 4.98% (equal to peak1 divided by peak5 three times), which is close to the feeding ratio (5.00%). This small difference is attributed to the similar reactivity of AM and DAAM in radical polymerization [48]. Furthermore, the weight average molecule weight (Mw) and polydispersity index (PDI) of the copolymers were determined via gel permeation chromatography (GPC) (Table S1). Although the Mw of PAD copolymers slightly increased with the growth of the DAAM feeding ratios (DAAM is larger than AM), the Mw and PDI values of all copolymers are close to PAM due to the same reaction temperature and time. Based on the approximately equal chain length, the comparison of PAD hydrogels was allowed for the selection of a suitable spinning dope component.

**Figure 2:**
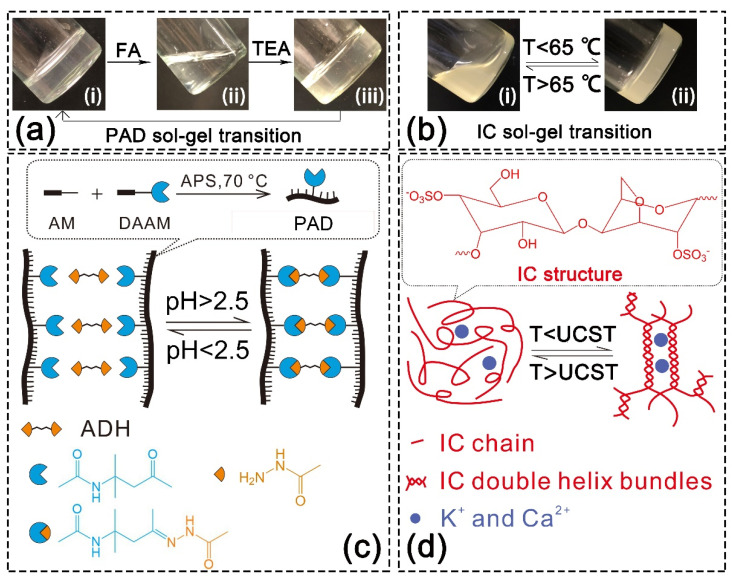
(**a**) pH-responsive sol-gel transition of PAD hydrogel: (**i**) original PAD-5 hydrogel crosslinked by ADH (pH = 6); (**ii**) hydrogel decomposed under acidic condition (pH = 1); (**iii**) hydrogel regenerated after pH neutralization (pH = 6); (**b**) temperature-responsive sol-gel transition of IC hydrogel: (**i**) IC solution formed above the UCST (upper critical solution temperature); (**ii**) IC hydrogel formed below the UCST; (**c**) scheme for pH-responsive sol-gel transition of PAD hydrogel (copolymer chain synthesis is illustrated in the dotted frame); (**d**) scheme for thermo-responsive sol-gel transition of IC hydrogel (IC structure is illustrated in the dotted frame).

Covalent SN hydrogels were synthesized using the keto-hydrazide reaction. The evidence of crosslinking is presented in Figure S2a. After adding adipic dihydrazide (ADH) into the aqueous solution of PAD-5, gelation was observed whether at reacting temperatures of 25 or 70 °C, and the crosslinking was accelerated by higher temperatures. Meanwhile, no gelation occurred in the parallelly incubated PAM and ADH mixture solution (Figure S2b). This phenomenon indicates that copolymer chains were crosslinked via a reaction between DAAM units and ADH. Specifically, ketone groups on the DAAM units reacted with the hydrazide groups on the ADH molecules to form acylhydrazone bonds (the reaction is diagramed in Figure S3). This bonding presented sensitivity to pH, since the adding of formic acid (which adjusted the pH to 1) and the neutralization by triethylamine (which adjusted the pH to 6) led to the decomposition and regeneration of PAD hydrogel, respectively (Figure 2a). The formation and breakage of acylhydrazone bonds were controlled by the pH (critical value = 2.5 [49]) and was allowed to be repeated (Figure 2c).

**Figure 3:**
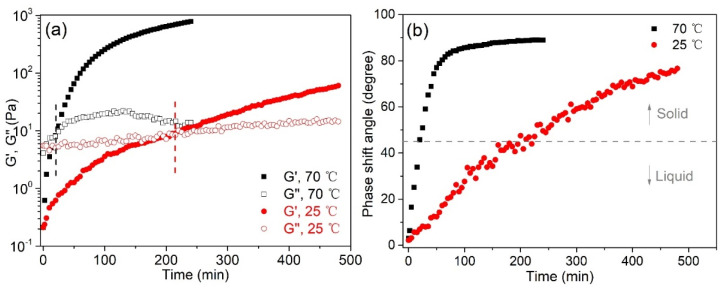
(**a**) G′ and G″ and (**b**) phase shift angle of PAD-5 hydrogel during the gelation process at 25 and 70 °C.

The gelation time is crucial for the spinning of hydrogels fiber; thus, the gelation process of PAD SN hydrogels at different temperatures was monitored through dynamic time sweep rheological experiments. As presented in Figure 3a, the storage modulus (G’) increased with the reaction time, and the growth trend at 70 °C is significantly higher than that at 25 °C. Additionally, the increasing of the phase shift angle followed the same trend (Figure 3b), indicating that the PAD precursor transferred from a liquid into a solid more rapidly at a higher temperature. The gelation times were recorded where crossovers of the storage modulus and loss modulus (phase shift angle = 45 degree) appeared. At a higher temperature, the gelation time shifted earlier (215 min at 25 °C and 20 min at 70 °C) due to the acceleration of the keto-hydrazide reaction. The kinetics of gelation were accelerated approximately ten times faster at 70 °C, indicating the faster equilibrium of acylhydrazone formation and transamination when the motions of PAD segments and ADH became intensified [50]. Such rapid equilibrium kinetics is desirable in the self-healing of damaged networks, where the rapid repairing of cracks by reformed acylhydrazone linkages is allowed [51]. Therefore, the gelation time of PAD SN hydrogel is controllable by manipulating the reaction temperature, in which 70 °C was chosen for further rapid and in situ hydrogel fiber preparation.

Physical SN hydrogel was prepared by cooling down IC aqueous solution. IC is a typical thermo-sensitive polysaccharide. As demonstrated in Figure 2b, the IC solution crosslinked into hydrogel when the temperature dropped below 65 °C, while the hydrogel decomposed into the solution when the temperature rose above 65 °C. This sol-gel transition is attributed to the thermo-sensitivity of IC (Figure 2d) [8]. When the temperature dropped below the upper critical solution temperature (UCST), the IC chains self-assembled to double-helix bundles via the hydrogen bonds. Then, the negatively charged sulfonate groups on the IC bundles and chains associated with cations via electrostatic attraction. These two supramolecular interactions caused the gelation of IC and were able to offer sacrificial bonds in the double-network structure. When the temperature rose above the UCST, the physical interactions disassociated and led to the decomposition of hydrogel.

**Figure 4:**
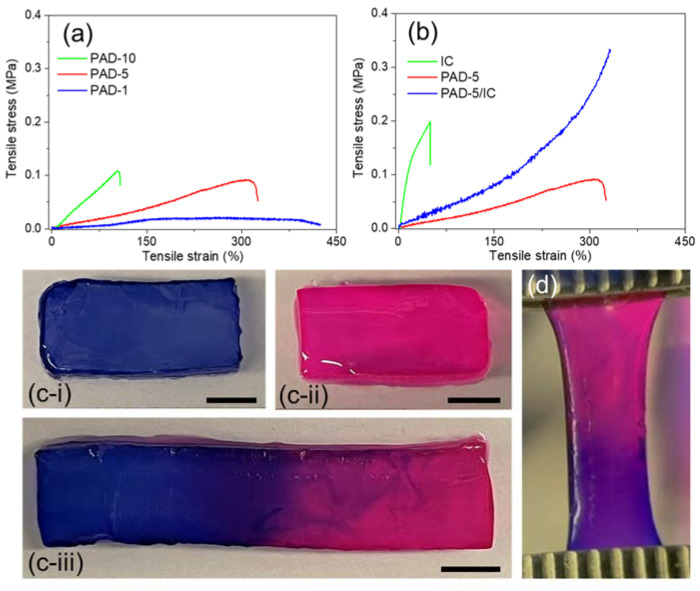
(**a**) Typical tensile curves of PAD SN hydrogels; (**b**) tensile curves of PAD-5, IC SN and PAD-5/IC DN hydrogels; (**c**) self-healing of PAD-5/IC DN hydrogel segments: rectangle hydrogels stained by (**i**) methylene blue and (**ii**) rhodamine B, (**iii**) self-healed hydrogel segment (interface smeared by formic acid, sealed and incubated at 70 °C for 4 h, all scale bars = 5 mm); (**d**) healed hydrogel showed stretchability.

Tensile tests were performed on PAD SN hydrogels to nominate a suitable copolymer in further studies. As presented in Figure 4a, PAD SN hydrogels presented higher strength but lower stretchability with the increase in DAAM contents, which decided the crosslinking density in these hydrogels. In comparison with PAD-1 (too weak) and PAD-10 (too brittle) hydrogels, PAD-5 hydrogel was nominated for further preparation of DN hydrogel due to its great toughness.

### 2.2. Tensile Properties and Self-Healing Performances of DN Hydrogels

To assess the feasibility of spinning, PAD-5/IC DN hydrogels were synthesized and compared with their parent SN hydrogels. As illustrated in Figure 4b, PAD-5 SN hydrogels presented a stretchable (ε~300%) but weak (σ < 0.1 MPa) performance, while IC SN hydrogels showed a stiff (*E*~1 MPa) but brittle (ε~50%) behavior, with an obvious yielding point. This yielding performance is attributed to the destruction of electrostatic attraction between the sulfonate groups and calcium ions [52]. The PAD-5/IC DN hydrogels exhibited significantly higher strength and stretchability due to the energy dissipation of sacrificed IC physical network. Moreover, PAD-5/IC hydrogels presented increased modulus at a highly stretched state, indicating the successful transition of the brittle facture to a ductile type [23]. At the highly stretched stage, polymer strands were aligned, which allowed the formation of highly oriented conformation during spinning.

Since both parental SN hydrogels presented stimuli-responsive sol-gel transitions, the PAD-5/IC DN hydrogel should be self-healable. Given that the sequential healing of two networks is time consuming, simultaneous healing is preferred in real applications. As demonstrated in Figure 4c, two differently colored DN hydrogel rectangles were joint together after cutting. By smearing formic acid on the interface and incubating at a temperature above the UCST of the IC, the joint segments completely healed into one rectangle, which was able to be stretched (Figure 4d) [53]. The self-healing process at the interface between two segments was recorded by optical microscopy (Figure S4a–c). With the increase in the healing time, the gap width between two segments decreased from 400 μm initially to 100 μm at 2 h. The disappearing of the crack was observed at 4 h of healing time, which indicated the near-complete self-healing of hydrogel segments. The spread of dye molecules between two segments led to a purple color at the boundary after healing, hinting the exchange of materials (including reaction partners) at the interface [50].

### 2.3. Spinning, Tensile Properties, Self-Healing Efficiencies and Shape Memory of DN Hydrogel Fibers

**Figure 5:**
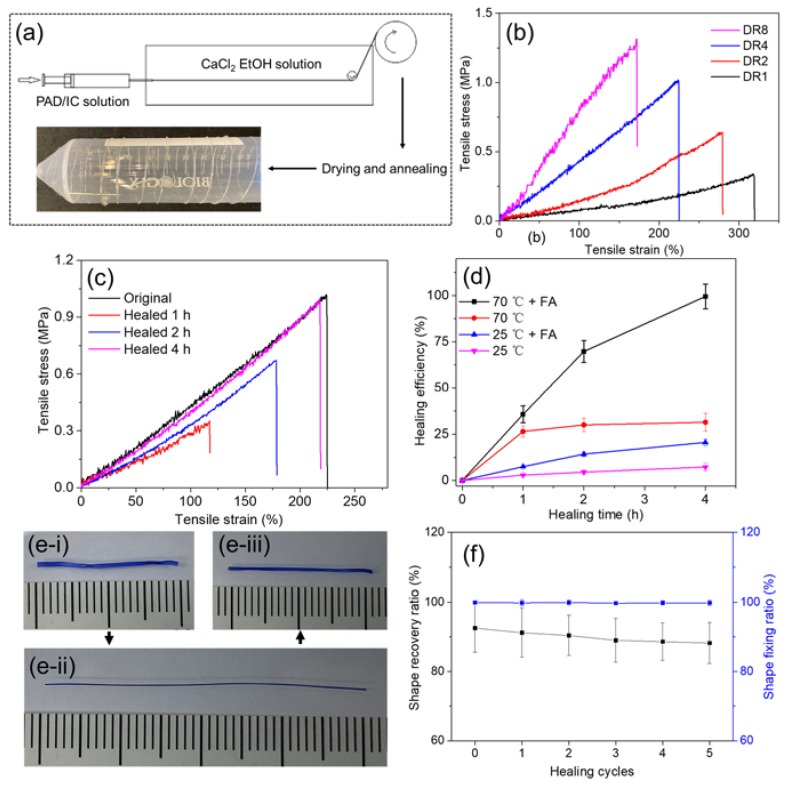
(**a**) Scheme of wet-spinning process of PAD-5/IC DN hydrogel fibers (inset photo: DR1 fiber winded on a plastic tube); (**b**) typical tensile curves of PAD-5/IC DN hydrogel fibers with increased DR; (**c**) typical tensile curves of PAD-5/IC-DR4 fibers before and after healing for 1, 2 and 4 h; (**d**) effect of healing time, temperature and formic acid on healing efficiencies; (**e**) recovery process of PAD-5/IC-DR4 SMF at states of (**i**) water swelled, (**ii**) post-drawn and dried, (**iii**) shape recovered; (**f**) shape recovery and fixing ratios of PAD-5/IC-DR4 SMF as a function of healing cycles.

Based on the PAD-5/IC hydrogel, DN fibers could be fabricated by wet spinning. A stable and flowable spinning dope is essential for continuous spinning. As a result, the pH of PAD-5/IC hydrogel precursor was adjusted to 1 in order to prevent covalent crosslinking before spinning, while the temperature of the syringe was retained at 70 °C to avoid physical gelation. To reduce the effect of the acidic hydrolysis of IC, the spinning dope was immediately used after it was obtained. The wet-spinning process is demonstrated in Figure 5a. Under the flowable state, the spinning dope was injected into the coagulation bath (10 wt% of CaCl_2_ ethanol solution). Through the electrostatic attraction between the calcium divalent cations and the negatively charged sulfonate groups on the IC [54], the dope column rapidly crosslinked into the nascent fiber and was progressively strengthened by the coagulation bath due to the continuous penetration of cations and the dehydration from the ethanol. During the spinning, the pH of the nascent fiber was neutralized via the diffusion of protons. Although the majority of the ADH was not reacted with the PAD, the loss of the crosslinker in the coagulation bath was negligible due to the dissolvability of the ADH in ethanol. After being collected on roller, the fibers were dried at 70 °C to remove the residual formic acid and annealed for higher crystallinity and dimensional stability.

During spinning, nascent fibers were stretched for a higher orientation degree. The influence of drawing ratios (DR) on the tensile properties and morphology was investigated. As presented in Figure 5b and summarized in Table 1, fibers with increased DR became stronger and stiffer, although the stretchability was decreased. These changes evidently indicated that the polymer strands in the highly stretched fibers became more aligned along the longitude direction, which boosted the fiber strength. The cross-section images of the fibers also showed that the fibers became thinner with the increase in the DR (Figure S5). Additionally, the swelling capacity of fibers was slightly affected by the aligned structure, which led to the restricted expansion of the network along the longitudinal direction as it absorbed water [55]. The least swelled fiber reached more than 10 times the swelling degree at the equilibrium state, which already meets the requirement of biomedical applications [14,56]. The toughness of the PAD-5/IC-DR4 fiber reached an attractively high value of 1.22 MJ/m^3^, which is suitable for applications in real products.

Since the self-healing performance was observed in the PAD-5/IC bulk hydrogels, the healing efficiency of the toughest DR4 fiber was quantitively evaluated by tensile tests (Figure 5d). After immersing the breaking ends of the failed fiber into formic acid and attaching the two ends together, the healing sample was sealed and incubated at 70 °C for 1, 2 and 4 h. In comparison with the original fiber, the healing fiber showed gradually enhanced strength and modulus with increasing time. The 4 h healed fiber reached 1.03 MPa of strength, which almost equaled that of the original fiber (giving it a 96% healing efficiency). Although the covalent and physical networks healed simultaneously, their respective contribution to the healing was studied by healing under different conditions (Figure 5d). At 70 °C, with formic acid smeared on interface, the fiber achieved highly efficient and rapid healing. The healed fiber at 25 °C without formic acid showed almost no healing (<15%). Without raising the temperature, the supramolecular network was not healed, but the dynamic regeneration of the acylhydrazone bonds contributed approximately 20% of healing at 4 h. Similarly, without the assistance of formic acid, although the fiber rapidly healed its supramolecular network in 1 h, the invalidation of the covalent network led to a low efficiency of <30% at 4 h. Benefiting from the convenient processing of fiber healing, such self-healable and super-tough DN hydrogel fibers may be applied in healable fabrics and flexible actuators.

As a potential shape memory fiber (SMF) with self-healing properties for actuating uses, the PAD-5/IC-DR4 fibers were swelled (to reach 90% of water content, Figure 5e-i) and post-drawn to 100% of strain. By drying under room conditions, the stretched shape of the fibers were fixed without obvious contraction after releasing the stretching (Figure 5e-ii). When the fixed SMFs were rehydrated in water, their glass transition temperature kept decreasing with the increase in water content. Water acted as plasticizer and led to the shape recovery of the SMFs (Figure 5e-iii), which started contracting in the longitudinal direction when their glass transition temperature dropped below the water temperature. Furthermore, by cutting the recovered fibers and healing them under optimal conditions (70 °C with acid), the shape recovery processes were repeated for five cycles. The shape fixing and recovery ratios of SMFs were determined in Figure 5f. With number of healing cycles increased from zero to five, the shape fixing ratios remained the same level of almost 100%. The shape recovery ratios kept decreasing from 92.5% (virgin SMFs) to 88.2% (healed five times), indicating the good repeatability and fatigue resistance of highly healed SMFs during multiple healing and recovering processes [57].

## 3. Conclusions

In summary, PAD copolymers with different monomer molar ratios were successfully synthesized via radical polymerization. The structure of PAD-5 was characterized by FTIR, NMR and GPC. The ketone groups on the DAAM allowed the formation of dynamic acylhydrazone bonds with the ADH, which led to the gelation of PAD SN hydrogels. The gelation time at 70 °C was determined through time sweep experiment evaluation. The IC SN hydrogels were prepared by facile cooling down. The PAD and IC hydrogels presented reversible self-healing performance in response to pH and temperature, respectively. Tensile studies on the PAD, IC and PAD/IC hydrogels indicated the construction of DN hydrogels and a nominated composition of spinning dope. The simultaneous healing of both networks in the PAD-5/IC hydrogel was achieved by heating and formic acid smearing on the interface. Via facile wet spinning, PAD-5/IC hydrogel fibers with DR ranging from 1 to 8 were fabricated. Such fibers reached a high strength of 1.35 MPa or a high toughness of 1.22 MJ/m^3^. The effects of healing time, temperature and formic acid on the healing efficiencies were studied with a tensile test. DR4 fiber was able to achieve highly efficient and rapid healing to reach 96% of its original strength in 4 h. The PAD-5/IC fibers were applied as SMFs for actuating in response to water. The highly efficient self-healing property of SMFs allowed the shape memory process to be repeated five times, indicating that these super-tough and self-healable DN hydrogel fibers may be ideal candidates in smart textiles.

## 4. Materials and Methods

### 4.1. Materials

AM (>99.9%), IC (>99%, 4.48 wt% K^+^ and 2.68 wt% Ca^2+^, tested via inductively coupled plasma mass spectrometry), calcium chloride (>99%) and APS (>99%) were purchased from Sigma-Aldrich Co (Shanghai, China). DAAM (>98%) and ADH (>99%) were purchased from Tokyo Chemical Industry Co., Ltd. (Shanghai, China). All chemicals were used as received without purification.

### 4.2. Synthesis of PAM and PAD

AM, DAAM and APS (0.31 mol% of monomers) were sequentially dissolved in degassed water at 70 °C. After 8 h of stirring (70 °C, 150 rpm, N_2_ degassing), copolymers were precipitated with acetone (equivalent mass of obtained mixture) and vacuum dried (50 °C, 24 h) (yield: 98.3%). The copolymers were denoted as PAD-n, where n stands for the molar percentage of DAAM. The unit contents of PAD-5 were evaluated by a ^1^H-NMR; ^1^H-NMR (400 MHz, D_2_O, r.t.) δ 1.28 (s, 6H, (CH_3_)_2_C(NH-)CH_2_-), 1.34–1.83 (br, 2H, -CH_2_- of the main ethylene chain), 1.91–2.36 (br, 1H, -CH- in the main ethylene chain), 2.14 (s, 3H, CH_3_-CO), 2.79–3.31 (br, 2H, CH_3_-CO-CH_2_-). PAM was parallelly synthesized with an equivalent monomer molar concentration (yield: 97.8%). The Mw and PDI (equal to Mw/Mn) of the synthesized polymers were determined via GPC (water mobile phase, reference: polyethylene oxide, 1260 ALS, Aglient, Santa Clara, USA).

### 4.3. Preparation of PAD, IC and PAD/IC Hydrogels

PAD hydrogel precursors were obtained by dissolving ADH (75 mol% of DAAM) in PAD aqueous solutions and rapidly injecting the mixture into a Teflon model (incubated at 25 or 70 °C for 8 h) to prepare covalent SN hydrogels. The time sweep experiments (frequency = 1 Hz, strain = 1.0%) were parallelly performed using a rheometer (MCR702, Anton Paar Inc,. Graz, Austria) with a flat plate of 25 mm diameter and a gap of 1 mm to evaluate the gelation time of the PAD-5 hydrogel. After the precursor solution was transferred onto the parallel plate (silicone oil was used to avoid water evaporation during the test) and reached the predetermined temperature, the G′, G″ and phase shift angle as functions of time were recorded. The gelation time was recorded when G′ = G″. The IC hydrogel precursor was obtained by dissolving IC and CaCl_2_ (75 mol% of sulfate groups) in water at 70 °C and rapidly injecting the mixture into a Teflon model until cooling down to room temperature. The PAD/IC DN hydrogel precursor was prepared after nominating PAD-5 as an optimized copolymer for further study. PAD-5 and IC precursors were mixed to obtain a homogeneous solution (PAD:IC = 3:1 *w*/*w*), and then rapidly injected into a Teflon model (incubated at 70 °C for 8 h). The water contents of all hydrogel specimens were fixed at 92.0 wt%.

### 4.4. Spinning of PAD/IC Hydrogel Fibers

PAD/IC hydrogel fibers were fabricated using a wet-spinning method. The homogeneous spinning dope was obtained by dissolving ADH (75 mol% of DAAM) in the degassed and acidic (pH = 1, adjusted by formic acid) aqueous solution that contained PAD (6.0 wt%) and IC (2.0 wt%). The dope (maintained at 70 °C) was injected into a coagulation bath (10.0 wt% of CaCl_2_ ethanol solution, 30 cm long, room temperature) using a syringe pump (spinning speed = 0.36 m/min, 18 G of spinneret, LSP01-1A, Baoding Longer Precision Pump Co., Ltd., Baoding, China). The solidified nascent fibers were collected on a drum with different winding rates and were monitored by a contact tachometer (DT-2236, Lutron Electronic Enterprise Co., Ltd., Taipei, China). The drawing ratios (DR, winding rate versus spinning speed) of nascent fibers were controlled as 1, 2, 4 and 8. All nascent fibers were oven dried (70 °C, 8 h) and annealed.

### 4.5. Fabrication and Shape Recovery Study of PAD/IC SMFs

Swelled PAD-5/IC-DR4 hydrogel fibers (to reach 90% of water content) were stretched to 100% of strain and air dried under stretching to obtain shape-fixed SMFs (fixed strain = ε_m_). SMFs were immersed into water at 20 °C to observe shape recovery performance. The residual strain (ε_r_) of recovered fibers was determined when recovery was completed. The shape fixing ratio was calculated by equation: R_f_ = ε_m_/stretched strain × 100%. Shape recovery ratios were calculated by equation: R_r_ = (ε_m_ − ε_r_)/ε_m_ × 100%. Recovered fibers were cut and healed (by smearing formic acid at the interface and maintaining them at 70 °C for 4 h). Shape recovery experiments were repeated on healed SMFs for 5 healing cycles to evaluate repetitive shape memory performance.

### 4.6. Characterizations

The chemical structures were characterized via ^1^H-NMR spectroscopy (Bruker Avance-III 400 MHz, Zurich, Switzerland). Chemical shifts were referenced to the solvent values (δ = 4.79 ppm for D_2_O). The chemical structure of each specimen was determined by FTIR (Perkin Elmer spectrum 100, Waltham, Massachusetts, USA) using wavenumbers between 4000 and 650 cm^−1^ with a 4 cm^−1^ resolution (spectra between 1630 and 1830 cm^−1^ were presented). Tensile properties were tested using a uniaxial tensile machine (Instron 4411, Norwood, MA, USA) with a 10.0 N load cell at an extension rate of 50.0 mm/min at 20.0 °C and 65.0% of relative humidity. The starting distance between two clamps was set as 20.0 mm. The tensile strength (σ, kPa) and stretchability (ε, %) were recorded at failure point. Young’s modulus (*E*, kPa) was calculated by fitting the initial linear region of stress–strain curve (tensile strain = 5~10%). The toughness (Γ, MJ/m^3^) was calculated according to the integral area of the tensile curves (MPa over mm/mm). The healing efficiency was calculated according to the percentage of the healed sample strength versus the original fiber strength. All tensile hydrogel samples were prepared in Teflon molds with a dumb-bell shape (50.0 × 4.0 × 2.5 mm^3^). All tensile properties were reported as mean and standard deviation (sample size = 6).

Hydrogel specimens were immersed into DI water until equilibrium swelling, and their weights (W_e_) were then measured. After completely oven drying them, the weights of dry hydrogels (W_d_) were recorded. The swelling capacities (Q) were calculated by expression: Q = (W_e_ − W_d_)/W_d_.

## Figures and Tables

**Table 1:** Summary of tensile properties and swelling capacities of PAD-5/IC hydrogel fibers (standard deviation in bracket, sample size = 6).

PAD-5/IC Fibers	Tensile Properties				Swelling Capacity (%)
	σ (MPa)	ε (%)	*E* (MPa)	Γ (MJ/m^3^)	
DR1	0.32 (0.02)	332.7 (23.99)	0.11 (0.01)	0.56 (0.08)	1235 (11)
DR2	0.66 (0.09)	281.6 (16.9)	0.39 (0.02)	0.82 (0.09)	1121 (7)
DR4	1.07 (0.05)	225.4 (15.8)	0.58 (0.04)	1.22 (0.10)	1087 (10)
DR8	1.35 (0.12)	168.4 (12.5)	0.81 (0.06)	1.09 (0.09)	1030 (9)

## Data Availability

The data presented in this study are available in the article.

## Supplementary Materials

The following supporting information can be downloaded at: https://www.mdpi.com/article/10.3390/gels8020101/s1, Figure S1: Scheme of radical polymerization of PAD; Figure S2: (a) Synthesis of PAD hydrogel; (i) PAD aqueous solution; (ii) crosslinked PAD SN hydrogel. (b) No gelation occurred in PAM solution; (iii) PAM aqueous solution; (iv) PAM and ADH mixture solution (scale bar = 1 cm). Figure S3: Crosslinking reaction between PAD chains and ADH. Figure S4: Interface cross-section images of PAD-5/IC hydrogel segments with healing time of (a) 0, (b) 2 and (c) 4 h (dye stained: above by rhodamine B, below by methylene blue). Figure S5: Cross-section images of (a) DR1, (b) DR2, (c) DR4 and (d) DR8 PAD-5/IC fibers. Table S1: Molecular weight (Mw) and polydispersity of synthesized polymers determined by GPC.
