# Supplementary material for: Self-Healable and Super-Tough Double-Network Hydrogel Fibers from Dynamic Acylhydrazone Bonding and Supramolecular Interactions
Source: Gels. 2022 Feb 8;8(2):101. doi: 10.3390/gels8020101 (PMC8871786; doi:10.3390/gels8020101)
Supplement: Supplementary file 1 [file gels-08-00101-s001.zip › gels-1560865-supplementary.pdf]

Supplementary

Self-Healable and Super-Tough Double-Network Hydrogel Fibers from Dynamic Acylhydrazone Bonding and Supramolecular Interactions

Jiachuan Hua [1], Chang Liu [1], Bin Fei [1,*] and Zunfeng Liu [2]

[1] Institute of Textiles and Clothing, The Hong Kong Polytechnic University, Hong Kong 999077, China; jc.hua@connect.polyu.hk (J.H.); judyc.liu@connect.polyu.hk (C.L.)
[2] Key Laboratory of Functional Polymer Materials, College of Chemistry, Nankai University, Tianjin 300071, China; liuzunfeng@nankai.edu.cn
* Correspondence: bin.fei@polyu.edu.hk

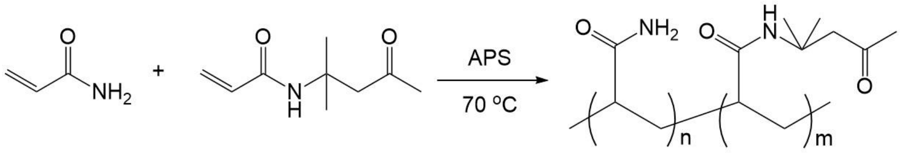

Figure S1. Scheme of radical copolymerization of PAD.

Table S1. Molecular weight (Mw) and polydispersity of synthesized polymers determined by GPC.

Polymers	DAAM feeding ratio (mol%)	Mw (kDa)	PDI
PAM	0	220	1.57
PAD-1	1.0	235	1.62
PAD-5	5.0	239	1.61
PAD-10	10.0	243	1. 64

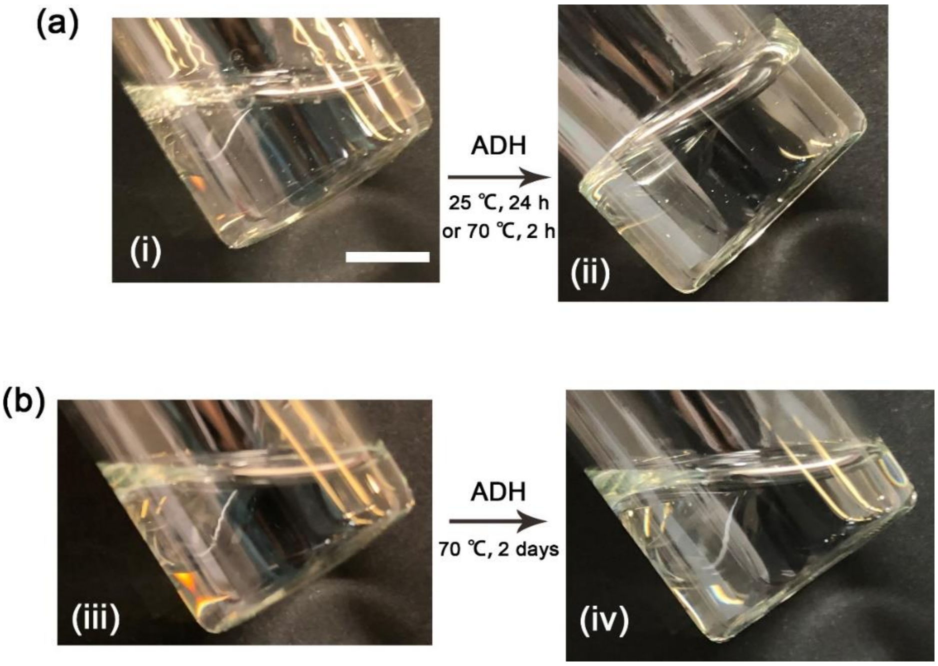

Figure S2. (**a**) Synthesis of PAD hydrogel; (**i**) PAD aqueous solution (6 wt%); (**ii**) crosslinked PAD SN hydrogel. (**b**) No gelation occurred in PAM solution; (**iii**) PAM aqueous solution (6 wt%); (**iv**) PAM and ADH mixture solution (All images share the same scale bar of 1 cm).

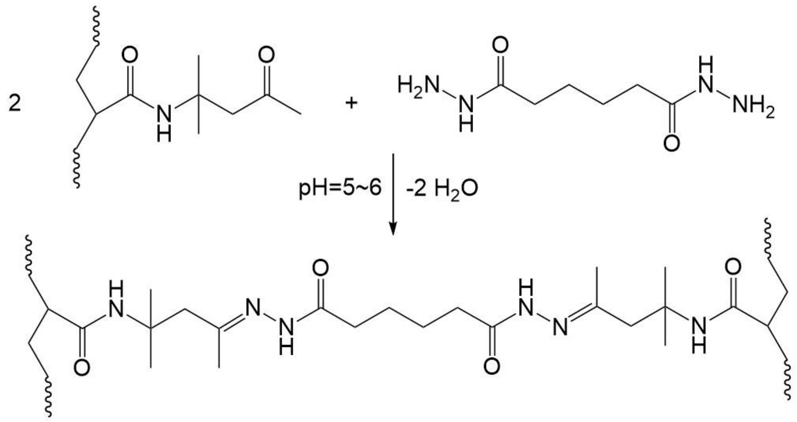

Figure S3. Crosslinking reaction between DAAM units on PAD and ADH (pH condition correspond to reaction in Figure S2a).

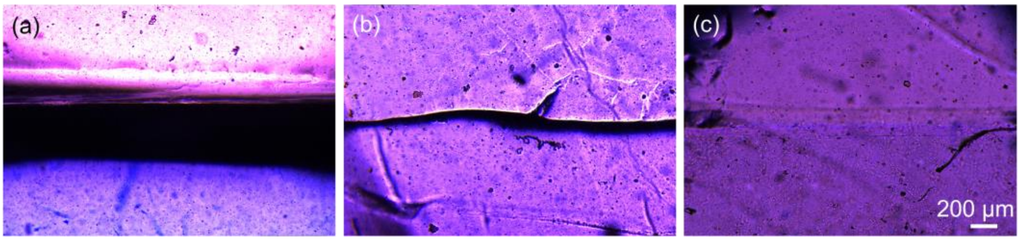

Figure S4. Interface crosssection images of PAD-5/IC hydrogel segments with healing time of (**a**) 0, (**b**) 2 and (**c**) 4 hours (dye stained: above by rhodamine B, below by methylene blue).

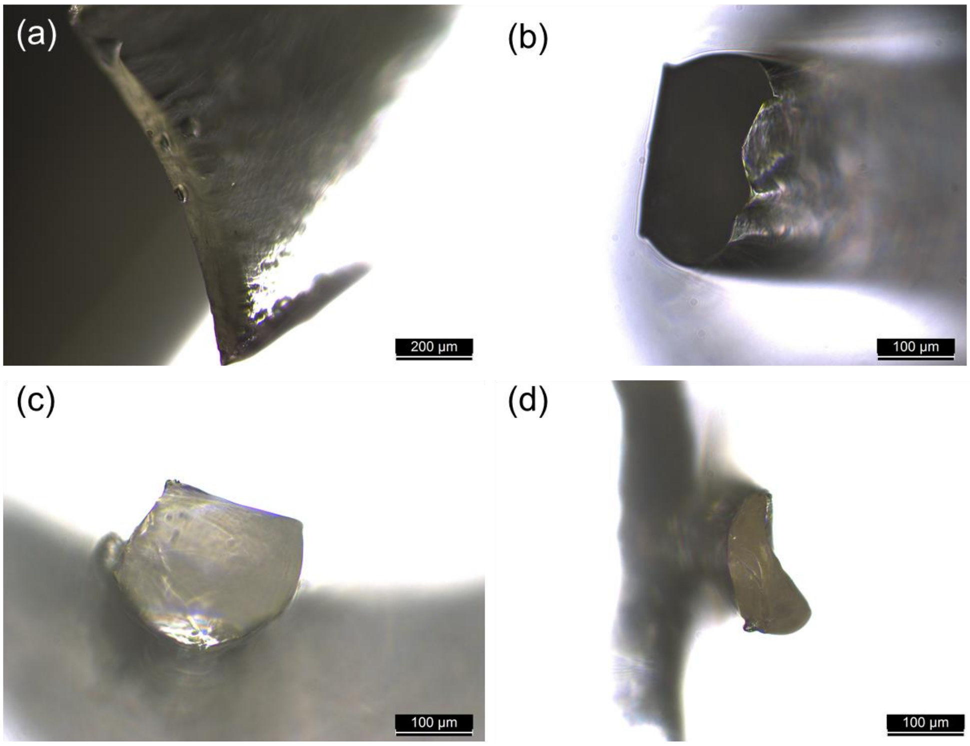

Figure S5. Crosssection images of (**a**) DR1, (**b**) DR2, (**c**) DR4 and (**d**) DR8 PAD/IC fibers.
